# Oncoplastic Breast-Conserving Surgery for Synchronous Multicentric and Multifocal Tumors: Is It Oncologically Safe? A Retrospective Matched-Cohort Analysis

**DOI:** 10.1245/s10434-021-10800-w

**Published:** 2021-10-06

**Authors:** Francesca De Lorenzi, Francesco Borelli, Eleonora Pagan, Vincenzo Bagnardi, Nickolas Peradze, Barbara Alicia Jereczek-Fossa, Cristina Leonardi, Giovanni Mazzarol, Giorgio Favia, Giovanni Corso, Emilia Montagna, Mario Rietjens, Paolo Veronesi

**Affiliations:** 1 Department of Plastic and Reconstructive Surgery, European Institute of Oncology, IRCCS, Milan, Italy; 2 Department of Statistics and Quantitative Methods, University of Milan-Bicocca, Milan, Italy; 3 Department of Breast Surgery, European Institute of Oncology, IRCCS, Milan, Italy; 4 Division of Radiotherapy, European Institute of Oncology, IRCCS, University of Milan, Milan, Italy; 5 Department of Pathology and Laboratory Medicine, European Institute of Oncology, IRCCS, Milan, Italy; 6 Medical Senology, European Institute of Oncology, IRCCS, Milan, Italy

## Abstract

**Background:**

Oncoplastic surgery is a well-established approach that combines breast-conserving treatment for breast cancer and plastic surgery techniques. Although this approach already has been described for multicentric and multifocal tumors, no long-term oncologic follow-up evaluation and no comparison with patients undergoing mastectomy have been published. This study aimed to evaluate whether oncoplastic surgery is a safe and reliable treatment for managing invasive primary multicentric and multifocal breast cancer.

**Methods:**

The study compared a consecutive series of 100 patients with multicentric or multifocal tumors who had undergone oncoplastic surgery (study group) with 100 patients who had multicentric or multifocal tumors and had undergone mastectomy (control group) during a prolonged period. The end points evaluated were disease-free survival (DFS), overall survival (OS), cumulative incidence of local recurrence (CI-L), regional recurrence (CI-R), and distant recurrence (CI-D), all measured from the date of surgery.

**Results:**

The OS and DFS were similar between the two groups. The incidence of local events was higher in the oncoplastic group, whereas the incidence of regional events was slightly higher in the mastectomy group. These differences were not statistically significant. The cumulative incidence of distant events was similar between the two groups.

**Conclusions:**

To the authors’ knowledge, the current study provides the best available evidence suggesting that the oncoplastic approach is a safe and reliable treatment for managing invasive multifocal and multicentric breast cancers.

Currently, oncoplastic surgery (ONC) is a well-established approach that combines breast-conserving treatment for breast cancer^1,2^ and plastic surgery techniques.^3,4^ It permits wide excisions that prevent breast deformities by reconstruction of large resection defects.^5,6^

This approach has cosmetic advantages, previously described in the literature.^7,8^ Moreover, ONC is superior to traditional quadrantectomy or lumpectomy in terms of accurate tumor resection and free resection margins^9,10^ and definitively is useful in extending the indications for breast conservation even in synchronous multicentric and multifocal breast cancers.^11^ Therefore a number of patients may benefit from conservative treatment instead of mastectomy. In addition, if a contralateral reduction mammaplasty is performed to achieve similar breast size after a large quadrantectomy and reshaping, a “surgical screening” of the contralateral healthy breast may allow the diagnosis of occult cancers.^12,13^

Few data exist regarding ONC and synchronous multicentric and multifocal breast cancers in terms of oncologic long-term results.^14^ No publication has specifically investigated the outcomes of synchronous multicentric and multifocal breast cancers treated by oncoplastic surgery, although small cohorts of patients with synchronous multiple cancers are included in the published series.^15–20^A recent systematic review by Winters et al.^21^ aimed to compare clinical outcomes after breast-conserving surgery versus mastectomy for multifocal and multicentric cancers, collectively defined as multiple ipsilateral breast cancers. Most of the studies they included were historical and did not report on ONC**.** The authors concluded that the available studies were mainly of moderate quality, historical, and underpowered, with limited follow-up evaluation and biased case selection favoring breast conservation rather than mastectomy for low-risk patients. The evidence was inconclusive, weakening support for the St Gallen consensus^22^ and supporting a future randomized trial.

A prospective American cohort study (National Institutes of Health, American College of Surgeons Oncology Group Z11102) is in progress to evaluate clinical outcomes of multiple ipsilateral breast cancers treated by therapeutic mammaplasties.^23^

A survey of UK surgeons confirmed that 90 % would welcome a randomized trial evaluating the efficacy of breast conservation compared with mastectomy (± reconstruction) for multiple ipsilateral breast cancers.^24,25^ The MIAMI trial will open as a preliminary study to assess whether a sufficient number of eligible patients would be willing to accept a randomized intervention during a 15-month period. This feasibility phase will inform the main trial, which is powered using a 2 % non-inferiority margin on a predicted 5-year local recurrence rate of 2.5 %, between breast conservation and mastectomy.

This study aimed to investigate the oncologic safety of ONC for synchronous multifocal and multicentric cancers.

## Patients and Methods

We identified 100 consecutive patients who underwent an ONC (monolateral, bilateral) for primary invasive multifocal or multicentric breast tumors in the European Institute of Oncology (IEO), Milan, Italy, between 2000 and 2015 and were included in the institutional database. This database is updated weekly and based on web data collection systems used for internal multidisciplinary meetings.

All the patients included in the database received adjuvant whole-breast radiotherapy plus an additional boost dose to the tumor bed using 6-MV energy beams. In most cases, the radiation method consisted of three-dimensional conformal radiotherapy (3DCRT) with conventional fractionation, whereas in the most recent part of the current series, intensity-modulated radiotherapy (IMRT) with hypofractionation via the Tomotherapy Hi-Art System (by Accuray, Sunnyvale, CA) was given on the basis of high-quality evidence showing equivalence in terms of efficacy and safety between conventional and shortened schemes.^26^ Compared with patients who did not receive ONC, a single wider radiation field was preferred for boosting the tumor bed. These patients formed the study cohort.

The patients who received intraoperative radiotherapy with electrons (ELIOT) to the tumor bed only or as a boost were not included in this series. To have a homogeneous population, we also excluded patients with secondary tumors or local relapses, bilateral tumors, or tumor treated with neoadjuvant chemotherapy. Therefore, the patients included in the current study represented only a fraction of the women who benefited from oncoplastic procedures during the same period.

For each patient in the study cohort, we selected from the database a matched patient who had mastectomy for invasive multicentric or multifocal tumors (control cohort) during the same period. The variables used to make the randomly assigned matches were age at surgery (within 5 years), year of surgery (within 3 years), tumor size, multicentricity, and multifocality (each patient with multifocal disease was matched with a control patient who had multifocal disease, and each patient with multicentric tumor was matched with a control patient who had multicentric tumor). All the patients were Caucasian.

The candidates for the oncoplastic approach or mastectomy were elected by consensus of senior breast and plastic surgeons according to the following variables: ratio of tumor size to breast size, tumor location, and patient preferences. All the patients signed a written informed consent, and the study protocol was approved by our Institute’s Scientific Committee and Risk Management Decisional Unit.

Multicentric tumors were defined as involving different quadrants in the same breast or as interspaced by at least 5 cm of non-neoplastic histologically proven parenchima. Multifocality was defined as discrete neoplasms involving the very same quadrant.^27,28^

After surgery, all cases were discussed during the weekly multidisciplinary meeting attended by plastic and breast-dedicated surgeons, medical oncologists, radiation oncologists, and pathologists. The decision for adjuvant systemic treatment was made on the basis of biologic features, tumor staging, previous treatments, and comorbidities according to internationally approved and regularly updated guidelines.^29^ The same protocol of medical treatment was delivered to the two groups.

The clinical follow-up evaluation of the two groups was similar and planned every 6 months. A radiologic examination of the breasts was performed every year (including a bilateral untrasound and mammogram for the patients who underwent conservation and a monolateral mammogram for those who underwent mastectomy) or more frequently in the case of clinical suspicion. Liver and bone as well as biologic markers were checked every year.

### Statistical Methods

The end points evaluated were disease-free survival (DFS), overall survival (OS), cumulative incidence of local recurrence (CI-L), regional recurrence (CI-R), and distant recurrence (CI-D), all measured from the date of surgery.

The study defined DFS as the time from surgery to events such as relapse (including ipsilateral breast recurrence, invasive or *in situ*), appearance of a second primary cancer (including contralateral breast cancer, invasive or *in situ*), or death, whichever occurred first. The study defined OS as the time from surgery until the date of death (from any cause).

The CI-L, CI-R, and CI-D were defined respectively as the time from the date of surgery to a local recurrence, a regional recurrence, and a distant metastasis. The DFS and OS functions were estimated using the Kaplan-Meier method. The log-rank test was used to assess differences between groups.

The functions of the CI-L, CI-R, and CI-D curves were estimated according to methods described by Kalbfleisch and Prentice,^30^ taking into account the competing causes of recurrence. The Gray’s test was used to assess cumulative incidence differences between groups.^31^ The hazard ratios (HRs) for DFS and OS comparing the ONC patients (study group) and the matched control group were estimated with the Cox proportional hazards model. The HRs for CI-L, CI-R, and CI-D comparing the ONC patients (study group) and the matched control group were estimated with Fine and Gray’s proportional sub-distribution hazard model. Patient and tumor characteristics were reported with absoulte and relative frequencies and compared with the chi-square test. All analyses were performed with SAS software v. 9.4 (SAS Institute, Cary, NC, USA). All reported *P* values are two-sided.

## Results

In the study group, mobilization and advancement of glandular flaps were performed for 62 patients (62 %) at the side of quadrantectomies. A superior pedicled reduction mammoplasty was performed for 16 patients (16 %), and an inferior pedicled reduction mammoplasty was performed for 17 patients (17 %). A round-block approach was used for four patients (4 %), and an implant was placed in one patient (1 %).

The monolateral ONC approach, with no surgery performed in the contralateral healthy breast, was used for 36 patients (36 %). The most frequent mammoplasty of the not diseased breast was a reduction (56 patients, 56 %) followed by mastopexy (3 patients, 3 %). Breast resection with reshaping for benign disease was performed for two patients (2 %). Senior plastic surgeons performed the glandular reconstructions at the time of quadrantectomies.

In the control group, 62 patients (62 %) underwent nipple areola-sparing mastectomies, and 48 patients (48 %) underwent skin-sparing mastectomies. Immediate reconstruction was performed for 93 % of the patients, including direct-to-implant reconstruction (64 patients), temporary expanders (28 patients), and muscular flaps (1 patient). No surgery was performed in the contralateral healthy breast of 49 patients (49 %). A simultaneous risk-reducing mastectomy was performed for two patients, and a breast resection with reshaping for benign disease was performed for two patients. The most frequent mammoplasty of the not diseased breast was mastopexy (24 patients), followed by augmentation (16 patients) and reduction (4 patients). Table 1 presents the type of surgery performed in the two groups.

**Table 1:** Surgery characteristics

	Oncoplastic (study cohort) (*n* = 100)		Mastectomy (control cohort) (*n* = 100)	
	*n*	%	*n*	%
Plastic surgery				
No	0	—	7	7.0
Yes	100	100.0	93	93.0
Type of surgical procedures for oncolplastic surgery				
Grandular flaps	62	62.0		
Inferior pedicle	17	17.0		
Superior pedicle	16	16.0		
Round-block	4	4.0		
Other	1	1.0		
Type of plastic surgery for mastectomy				
No plastic surgery			7	7.0
Tissue expander			28	28.0
Prosthesis			64	64.0
Other			1	1.0
Contralateral surgery				
No	36	36.0	49	49.0
Yes	62	62.0	49	49.0
Unknown	2	2.0	2	2.0

All the patients in the ONC group with four or more positive axillary nodes received postoperative irradiation to the whole breast and to the infra/supraclavicular region. The most used regimen was conventional fractionation, consisting of 50 Gy to the whole breast plus an additional boost dose of 10 Gy to the tumor bed, delivered with 3DCRT. Since 2012, the hypofractionated 15-fraction scheme (2.67 Gy per fraction) plus simultaneous integrated boost (3.2 Gy per fraction) using IMRT has been applied.

The same schedules also were applied to the infra/supraclavicular region for patients with four or more positve nodes. Only 50 % of the patients who underwent mastectomy received postoperative locoregional irradiation, using either conventional fractionation (45–50.4 Gy in 25–28 fractions) or hypofractionation (40.05 Gy in 15 fractions) (100 % vs 50 %; *P* < 0.001) because of locally advanced stage (≥ positive axillary nodes, T3–T4, unfavorable histopathologic features).

With regard to the clinicopathologic features not included in the matching algorithm (menopausal status, histology, tumor size and grade, tumor subtype, perivascular invasion, and adjuvant systemic treatments), the two groups were well balanced. Table 2 summarizes the characteristics of the patients stratified for ONC and mastectomy (control group).

**Table 2:** Characteristics of patients according to study group

	Oncoplastic (study cohort) (*n* = 100)		Mastectomy (control cohort) (*n* = 100)		*P* Value
	N	%	N	%	
Year of surgery					^a^
<2010	45	45.0	42	42.0	
≥2010	55	55.0	58	58.0	
Age at surgery (years)					^a^
<35	8	8.0	6	6.0	
35–50	55	55.0	56	56.0	
51–65	28	28.0	30	30.0	
>65	9	9.0	8	8.0	
pT					^a^
pT1	59	59.0	59	59.0	
pT2	40	40.0	40	40.0	
pT3	1	1.0	1	1.0	
Multifocal/multicentric tumor					^a^
Multifocal	84	84.0	84	84.0	
Multicentric	16	16.0	16	16.0	
Menopausal status					0.46
Premenopausal	66	66.0	61	61.0	
Postmenopausal	34	34.0	39	39.0	
Histology					0.61
Ductal	77	77.0	80	80.0	
Lobular/mixed/other	23	23.0	20	20.0	
pN					0.23
pN0	45	45.0	42	42.0	
pN1	30	30.0	38	38.0	
pN2	15	15.0	7	7.0	
pN3	10	10.0	13	13.0	
Grade					0.54
1	11	11.0	16	16.0	
2	51	51.0	46	46.0	
3	34	34.0	36	36.0	
Unknown	4	4.0	2	2.0	
Subtype					0.54
Luminal A	40	40.0	43	43.0	
Luminal B	51	51.0	45	45.0	
HER2-positive	6	6.0	5	5.0	
Triple-negative	3	3.0	7	7.0	
Perivascular invasion					0.47
No	64	64.0	59	59.0	
Yes	36	36.0	41	41.0	
Radiotherapy					<0.001
No	0	0	50	50.0	
Yes	100	100.0	50	50.0	
Adjuvant systemic therapy					0.90
No adjuvant therapy	2	2.0	2	2.0	
ET	56	56.0	51	51.0	
CTx	9	9.0	11	11.0	
CTx+ET	33	33.0	36	36.0	

*HER2* human epidermal growth factor receptor 2, *ET* estrogen therapy, *CTx* chemotherapy

^a^Matching variables

The sentinel node biopsy was negative for 45 % of the ONC patients and 42 % of the control patients. In case of a negative sentinel node in the frozen section, no further axillary surgery was performed in either group. Only for one patient in the control group was level 1 dissection performed, although the sentinel node was negative, with suspicion of nodal involvement. Isolated tumor cells in the node were considered negative. Complete axillary dissection (3 levels) was performed for 55 % of the ONC patients and 58 % of the control patients. Four of the control patients underwent complete axillary dissection, and the node results were negative.

**Fig. 1:**
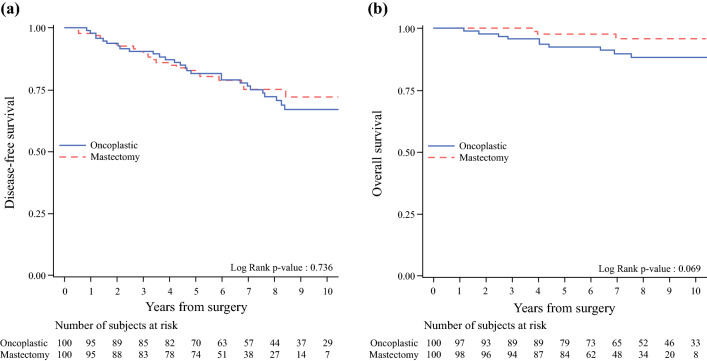
**a** Disease-freel and **b** overall survival according to study group

Table 3 describes the events in both groups, with an overall median follow-up period of 7.8 years (interquartile range [IQR], 5.7–9.7 years). The OS at 10 years was similar in the two groups (88.3 % in the ONC group and 95.8 % in the control group). The DFS at 10 years also was similar in the two groups (67.3 % in the ONC group and 72.2 % in the control group; Fig. 1).<F1> All local relapses were invasive cancers. The incidence of local events was slightly higher in the ONC group (8.2 % vs 2.2 % at 10 years) as expected, whereas the incidence of regional events was slightly higher in the mastectomy group (1.1 % vs 2.9 % at 10 years).

**Table 3:** Survival outcomes according to study group

	Oncoplastic (study cohort) (*n* = 100)	Mastectomy (control cohort) (*n* = 100)
Median follow-up: years (IQR)	9.2 (6.6–11.3)	7.1 (5.4–8.7)
OS		
Observed deaths: *n* (%)	10 (10.0)	3 (3.0)
Breast cancer related	6	2
Non-breast cancer-related	2	1
Unknown causes	2	0
5-Year OS (95 % CI)	92.5 (84.9–96.3)	97.8 (91.3–99.4)
10-year OS (95 % CI)	88.3 (79.1–93.6)	95.8 (87.1–98.7)
* P*-value (log-rank test)	0.069	
HR (95 % CI) [crude]	3.11 (0.86–11.3)	Ref.
DFS		
Observed events: *n* (%)	28 (28.0)	22 (22.0)
Local events	7	2
Regional events	1	2
Distant metastases	14	14
Other events	6	4
5-Year DFS (95 % CI)	81.8 (72.4–88.3)	81.8 (72.4–88.3)
10-Year DFS (95 % CI)	67.3 (55.6–76.5)	72.2 (59.5–81.5)
*P* value (log-rank test)	0.736	
HR (95 % CI) [crude]	1.10 (0.63–1.94)	Ref.
Cumulative incidence of local recurrence		
5-Year (95 % CI)	1.1 (0.1–5.2)	2.2 (0.4–6.9)
10-Year (95 % CI)	8.2 (3.3–16.0)	2.2 (0.4–6.9)
*P* value (Gray test)	0.262	
HR (95 % CI) [crude]	2.61 (0.58–11.9)	Ref.
Cumulative incidence of regional recurrence		
5-Year (95 % CI)	1.1 (0.1–5.5)	1.1 (0.1–5.2)
10-Year (95 % CI)	1.1 (0.1–5.5)	2.9 (0.5–9.5)
*P* value (Gray test)	0.499	
HR (95 % CI) [crude]	0.46 (0.04–5.43)	Ref.
Cumulative incidence of distant recurrence		
5-Year (95 % CI)	11.6 (6.1–19.0)	12.9 (7.0–20.7)
10-Year (95 % CI)	16.3 (9.2–25.2)	17.3 (9.2–27.4)
* P* value (Gray test)	0.882	
HR (95 % CI) [crude]	0.95 (0.46–1.99)	Ref.

*IQR* interquartile range, *OS* overall survival, *CI* confidence interval, *HR* hazard ratio, *DFS* disease-free survival

**Fig. 2:**
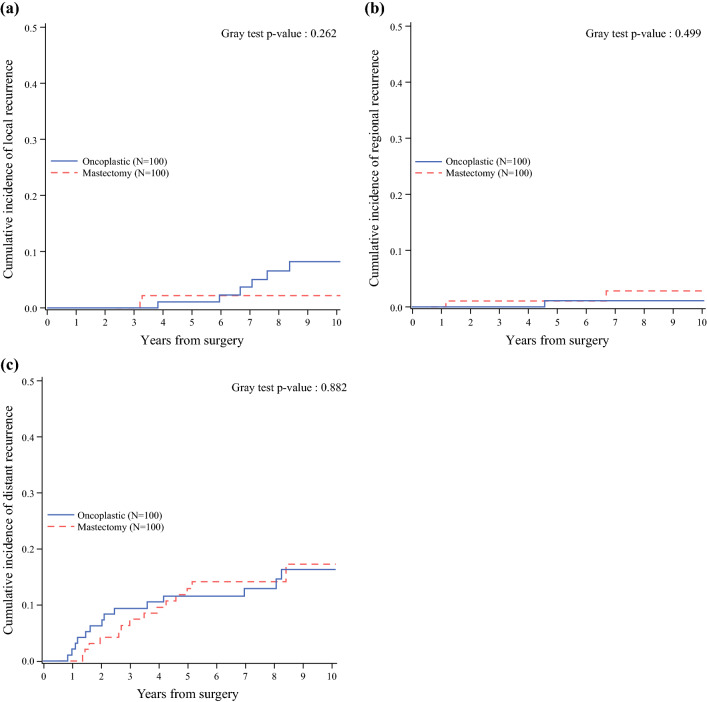
Cumulative incidence of **a** local recurrence, **b** regional recurrence, and **c** distant recurrence according to study group.

Regional events included axillary metastases (2 patients) and supraclavicular nodes metastases (1 patient). These differences were not statistically significant. The cumulative incidence of distant events was similar in the two groups (16.3 % in the ONC group vs 17.3 % in the mastectomy group at 10 years; Fig. 2). Distant metastases affected the bone (*n* = 10), liver (*n* = 6), lung (*n* = 1), and other or multiple sites (*n* = 11).

## Discussion

The results for our series of 100 consecutive oncoplastic procedures suggest the safety of ONC as an integral part of multicentric and multifocal cancer treatment. The two groups had highly similar DFS and OS. Because the two groups were matched by age, year of surgery, tumor size, multifocality, multicentricity, and prognosis, the patients followed a similar therapeutic protocol. Thus, the difference in OS or DFS could be explained by the different surgical approaches. We can therefore infer that the type of oncologic resection did not influence the clinical outcome of the disease.

As expected, we observed a small increase of local events in the ONC group, but without statistical significance, which did not influence the rates of either OS or distant events. Some authors have reported an inferior local control after breast-conserving surgery for multicentric lesions versus unifocal tumors,^32^ possibly due to a greater residual burden of microscopic neoplastic foci to be controlled with radiotherapy. Moreover, ONC increases the difficulty of localizing the original tumor bed due to the mobilization and displacement of glandular flaps, resulting in a potential geographic miss of the area considered at higher risk of relapse, especially in young women and grade 3 tumors. In addition, the tendency of enlarging the boost radiation field in the attempt to cover the original tumor sites can lead to a greater risk of fibrosis with an impaired cosmetic outcome.^33^

On the other hand, we observed a slight increase of regional events in the mastectomy group, with no impact on OS. The ONC group showed a lower rate of regional events. This finding could be related to the delivery of whole-breast radiotherapy, although the tangential fields normally provide a limited dosimetric coverage of the axilla contents. In fact, a dosimetric analysis of first axillary level coverage using standard radiation fields showed that only 1 in 15 patients had 35 % of the axillary volume enclosed in the 100 % isodose.^34^ However it seems that even a limited therapeutic dose for potential axillary residual disease derived from the tangential fields of the breast is sufficient to decrease the rate of axillary nodal relapse, as shown in the study comparing axillary recurrence between patients undergoing full-dose intraoperative radiotherapy and those undergoing whole-breast radiotherapy for stage T1N0 tumors.^35^

On the other hand, only 50 % of the patients who underwent mastectomy received postoperative irradiation. Currently, radiotherapy is indicated for patients with a tumor 5 cm in size or larger and at least two additional risk factors^36^ and for patients with at least four axillary positive nodes or internal mammary chain involvement. Patients with one to three involved nodes receive radiotherapy in case of adverse pathology according to the 2019 St Gallen Consensus.^37^

Because the study had the limitation of its retrospective nature, we could not collect relevant data about breast volume from the patients’ charts. We expected to find a larger mean breast volume among the patients who underwent ONC. In fact, we believe that the ratio of tumor size to breast size surely influences the necessity of mastectomy even if a combined oncoplastic approach can guarantee adequate free margins and satisfactory cosmetic results. The ONC approach might extend the indications for breast conservation for multifocal and multicentric tumors located in medium-large breasts. For smaller breasts, mastectomy still is the gold standard. Accordingly, multicentric tumors were more frequently treated by mastectomy in our series.

To our knowledge, the current study adds to the growing body of evidence supporting the benefits of ONC for multifocal and multicentric cancer patients conventionally treated with mastectomy, improving the evidence base for ONC. Recurrence and survival rates, the ultimate measures of oncologic safety, are satisfactory. The ONC approach is comparable with mastectomy. The two groups in this study were well balanced and received the same protocol of adjuvant treatments. The follow-up period was sufficiently long to detect the vast majority of adverse events.

We are aware that the major limitation of this study was that the data were collected retrospectively, whereas the follow-up information was supplied by a prospective database. We fully agree that we need future randomized clinical trials to definitely demonstrate the safety of ONC for multifocal and multicentric tumors and to answer questions still unanswered, such as the use of a double radiotherapy boost after double lumpectomies for multicentric tumors or biologic features on multiple ipsilateral breast cancers.
